# Sequential Diluted Dual-Curable Epoxy-Anhydride Thermosets: Effect of Epoxy-Based Reactive Diluent on Curing Behavior, Thermal and Mechanical Properties

**DOI:** 10.1021/acsomega.6c04947

**Published:** 2026-07-22

**Authors:** Anna M. Dlugaj, David May

**Affiliations:** † Leibniz-Institut für Verbundwerkstoffe GmbH, Erwin-Schröndiger 58, 67663 Kaiserslautern, Germany; ‡ Faserinstitut Bremen e.V., University of Bremen, 28359 Bremen, Germany

## Abstract

The main drawback of sequential dual-curable off-stoichiometric epoxy-anhydride thermosets diluted with 1,6-bis­(2,3-epoxypropoxy)­hexane (HDDGE), which is low glass transition temperature after final curing at 150 °C, was overcome by using 1,4-bis­(2,3-epoxypropoxy)­butane (DD) as a difunctional aliphatic epoxy-based reactive diluent. In addition to this diluent, diglycidyl ether of bisphenol A, tetrahydromethylphthalic anhydride and 1-methylimidazole were mixed and used as epoxy, anhydride comonomer, and anionic initiator, respectively. The quasi-sequential curing behavior of off-stoichiometric formulations arises from two reactions: the anhydride–epoxy condensation (esterification) and epoxy homopolymerization (etherification). Although these reactions are designed to occur sequentially, the overlap in their activation temperature ranges leads to partial competition between them. The relative contribution of both curing stages, as well as precuring time, determine the changes in viscosity during the curing process. It is therefore difficult to precisely separate these two curing stages, which makes dual-curing formulations quasi-sequential. The final off-stoichiometric material showed a *T*_g_ of 137.1 °C, determined from the peak temperature of the damping factor (tan δ) vs temperature curve obtained by Dynamic Mechanical Analysis, and the temperature at which the mass reduction of 5 wt % occurred under air atmosphere (*T*_–5wt–%_) was 365.6 °C, determined by Thermogravimetric Analysis. For comparison, the dual-curable off-stoichiometric epoxy-anhydride thermosets containing HDDGE showed a *T*_g_ of 89.0 °C and a *T*_–5wt–%_ of 343.3 °C. However, the use of DD as a diluent comes with the drawback that the developed off-stoichiometric formulations have a reduced mechanical strength of 65.5 MPa, which is 14% less than comparable off-stoichiometric thermosets containing HDDGE.

## Graphical abstract

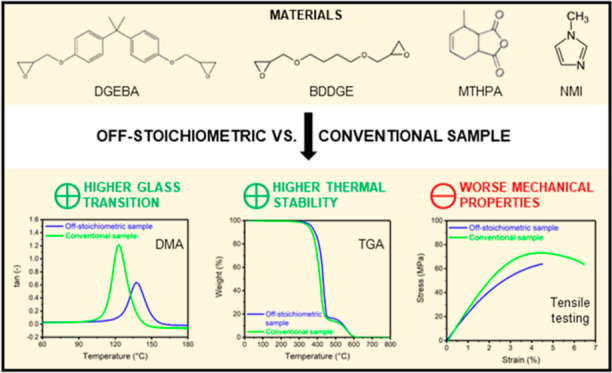

## 1 Introduction

Dual-curing refers to the combination of two different, compatible, and well-controlled polymerization reactions occurring simultaneously or sequentially.^1–5^ This method combines two otherwise distinct polymer networks to form an interpenetrating polymer network that exhibits superior properties to those of its individual parts.^6^ While simultaneous dual-curing aims at improving the final properties of thermosets,^3–6^ sequential dual-curing additionally aims at controlling the curing sequence in order to obtain materials with two different sets of properties after the first curing reaction (intermediate material) and the second curing reaction (final material).^5^ The relative contribution of both curing stages determines the viscosity changes throughout the curing process, i.e., the intermediate material can take a form ranging from liquid-like to rigid. This form can be preserved or destroyed by a spontaneous decrease in viscosity during the activation of the second curing stage. This type of processing allows for better control over the network structure build-up, offering the possibility of producing components with complex shapes.^2^ In the literature, a wide range of applications is proposed, from shape memory materials,^1,4,7,8^ through soft coatings for delicate substrates,^4,9^ impression materials,^7^ dry adhesive bondings,^10^ holograms,^11,12^ structured surface topologies,^13^ stereolitography,^14^ 3D-printable materials,^15–21^ to matrices in composite materials.^22–24^

Over the last couple of years, several requirements for successful sequential dual-curing have been formulated: (a) activation of both curing reactions by different stimuli (e.g., ultraviolet light, heat) or by sufficiently different reaction rates to separate the two curing reactions in terms of different curing conditions (e.g., time and temperature); (b) compatibility and selectivity of both curing reactions to avoid reactivity effects or undesired inhibition;^3–6^ and (c) the ability to tailor the properties of intermediate and final materials to individual applications.^3,5^ In addition to these criteria, Hajebi et al.^25^ have recently proposed five additional requirements for the curing agent that activates the first curing stage: (a) no interaction with the second curing stage or its activation; (b) high reaction rate; (c) yield of almost 100%; (d) simple curing conditions; and (e) no negative impact on the properties of the final materials. To this end, although many dual-curable thermosets based on combined thermal and photopolymerization reactions in a single thermosetting formulation have been reported in the literature (e.g., epoxy-acrylate,^17^ acrylate-based,^26^ or epoxy-based thermosets^27^), we focus on those based solely on two combined thermal polymerization reactions. The most commonly investigated systems are epoxy-based dual-curable thermosets, which can generally be created in two different ways. The first involves mixing an epoxy resin, a curing agent (e.g., thiol,^1,2,4^ amine,^28,29^ anhydride,^30^ or dicarboxylic acid^31^) in deficient amount and an accelerator (e.g., imidazole) to obtain off-stoichiometric epoxy-excess formulations. This causes some epoxy groups to react with all available reactive groups of the chosen curing agent at lower temperatures, while the remaining epoxy groups homopolymerize in the presence of an initiator only at higher temperatures.^1,2,4,28–31^ The second way involves mixing epoxy resin, two curing agents with different reactivities and optionally an accelerator.^22,23,32–36^ This causes some epoxy groups to react with all amine groups of the precuring or rather reactive curing agent (e.g., diethylenetriamine,^23^ *m*-xylylendiamine^36^) at lower temperatures, while the remaining epoxy groups react with all amine groups of the latent curing agent (e.g., Dicyandiamide,^23,36^ diaminodiphenylsulfone^32,33^ ) optionally in the presence of initiator (e.g., DIURON^23^) only at higher temperatures. Apart from epoxy-based thermosets, it is worth mentioning that the concept of sequential dual-curing has been investigated for epoxy-acrylate formulations containing thiol curing agent and an initiator. In this case, the reaction between the acrylate groups with some of the thiol groups occurs at lower temperatures, while the epoxy groups react with the remaining thiol groups only at higher temperatures.^37,38^ The sequentiality, even if not studied in detail, has also been observed in the case of phthalonitril-propargyl ether oligoimides. The first curing reaction here is the propargyl group curing with the formation of chromene and hydroxyl moieties, and the second reaction is the reaction between the phthalonitril units with the structures formed in the previous reactions.^39^

Despite the many possibilities for combining two thermal curing reactions in a single thermosetting formulation, there are also certain challenges. First, it is difficult to obtain formulations with ideal sequential dual-curing behavior; many dual-curable formulations still exhibit little or partial overlap between the two curing stages, resulting in the formation of unstable intermediate materials.^28^ These formulations can be called quasi-sequential because, despite their apparent sequentiality, it is difficult to precisely separate the two curing stages. In most cases, though, intermediate materials remain stable for a specified period of time at room temperature (RT) without significant changes,^37^ or can be safely stored at temperatures below their glass transition temperatures (*T*_g_).^30^ Second, the development of dual-curable formulations with the desired viscosity profiles can become a time-consuming trial-and-error approach. Even if it is possible to theoretically predict the gelation of dual-curable epoxy-thiol or epoxy-amine formulations, experimental observations often deviate due to lower functionality of curing agents caused by presence of impurities,^2,37^ or internal loop formation.^28^ Third, the majority of dual-curable formulations exhibit higher initial viscosity at RT compared to conventional formulations, since the increase in viscosity is an inevitable consequence of using a deficient amount of precuring agent. Although this characteristics may be an obstacle in many applications, this issue is rarely discussed in the literature. In our previous articles, we used an epoxy mixture of 75–90% diglycidyl ether of bisphenol A (DGEBA; epoxy resin) and 10–20% 1,6-bis­(2,3-epoxypropoxy)­hexane (HDDGE; diluent) to counteract this effect and obtain low viscous dual-curable epoxy-amine and anhydride-epoxy formulations. Nonetheless, the addition of HDDGE caused unexpected changes in curing behavior, which led to difficulties in postcuring, that is, the curing process was still not complete after final curing at 150 °C, resulting in lower final *T*_g_ values.^36,40^ In this article, research focuses on finding a diluent that does not cause these defects. Therefore, we first reviewed the literature on diluents used in epoxy resins.

Diluents, which are typically added to epoxy resin in amounts ranging from 2 to 20 wt %,^41^ are used to reduce viscosity in order to improve processability,^42,43^ increase the possible level of filler loading,^41,43,44^ reduce the cost of the formulation,^41,44^ or improve certain physical properties such as impact and peel strength.^44^ However, diluents weaken the interaction between the resin molecules,^42^ and can negatively impact properties such as *T*_g_, tensile strength or chemical resistance.^41^ Diluents can be classified into nonreactive and reactive types.^41–43^ Nonreactive diluents, which include solvents and plasticizers (e.g., benzyl alcohol,^41^ toluene, styrene), do not participate in the reaction between the epoxy resin and the hardener,^42^ and are therefore not chemically bound into the cross-linked network.^43^ The deterioration of properties occurs faster with increasing amount of nonreactive diluents than with increasing amounts of reactive diluents, which is why reactive diluents are preferred.^41^ Reactive diluents are capable of chemical reaction with epoxy resin,^43,45^ and can be divided into nonepoxy-based and epoxy-based.^43,46^ Nonepoxy-based reactive diluents, which include acrylates,^41,46^ polyols,^46^ triphenyl phosphite and lactone compounds,^43^ are more likely to leach-out, which is why epoxy-based reactive diluents are preferred.^46^ Epoxy-based reactive diluents contain at least one epoxy group^45^ that can chemically bind to the resin and form a part of the network after cross-linking.^42^ The most widely used reactive diluents are based on derivates of glycidyl ether,^44^ which can be classified into mono- (e.g., butyl glycidyl ether, phenyl glycidyl ether),^41,43^ di- (e.g., butanediol diglycidyl ether, neopentyl glycol diglycidyl ether),^44,46^ and polyfunctional (e.g., glycerol triglycidyl ether, pentaerythritol tetraglycidyl ether).^47^ Alternatively, glycidyl esters of acids,^41^ styrene oxide or olefin oxides,^45^ and epoxidized vegetable oils (e.g., epoxidized linseed oil,^46^ epoxidized soybean oil^48^) can be used. The limited number of articles on reactive diluents still does not provide sufficient guidance on how they function before, during and after cure.^49^ In general, however, polyfunctional epoxy-based reactive diluents are preferred over monofunctional diluents in order to preserve important mechanical and physical properties, because they have less impact on cross-link density, and, in some cases, may even improve certain properties.^43^

Taking all of the above-mentioned aspects into account, we propose a design concept for sequential dual-curable thermosets aimed at simultaneously achieving low viscosity and low reactivity at RT, as well as high *T*_g_ values of final materials. Our motivation is the potential that this research has to further expand the range of applications for dual-curing technology beyond its current main areas (such as shape memory or 3D-printable materials) toward the thermoset composite processing. In this context, we report the development of sequential dual-curable off-stoichiometric diluted anhydride-epoxy thermosets, in which low viscosity is ensured by the diluent and low exothermicity by the anhydride curing agent. Five epoxy-based diluents differing in their chemical structure, functionality and epoxy equivalent weight (EEW) were chosen to be added to the formulations consisting of DGEBA, tetrahydromethylpthalic anhydride (ANHY) and 1-methylimidazole (IMI) in order to identify the most suitable one that exhibits high *T*_g_ value, does not cause postcuring difficulties (unlike HDDGE) and does not drastically accelerate curing at RT. The effect of the diluent on the dual-curing character of anhydride-epoxy formulations, resulting from the occurrence of two competing reactions, was investigated. These are base-catalyzed anhydride-epoxy condensation (also known as esterification^50,51^ ) at lower temperatures, followed by homopolymerization of the epoxy excess (also known as etherification^50,51^ ) at higher temperatures, as described by Morancho et al.^30^ The most promising epoxy-based reactive diluent was then thoroughly investigated in terms of (a) curing behavior using differential scanning calorimetry (DSC) and Fourier-transform infrared spectroscopy (FTIR); (b) changes in the viscosity of intermediate materials during the activation of epoxy hompolymerization (etherification) observed in aluminum dishes; and (c) final properties using dynamic mechanical analysis (DMA), thermogravimetric analysis (TGA), tensile and three-point bending testing. For comparison purposes, the final properties of the conventional (nearly stoichiometric) formulation containing the most promising diluent were determined. This study is also significant inasmuch as it shows that certain important changes resulting from the presence of a diluent are observed exclusively in off-stoichiometric formulations, which may suggest that similar changes can be expected in other off-stoichiometric formulations (e.g., those containing a different curing agent) and/or that another additive could potentially cause different changes in off-stoichiometric formulations that do not typically occur in conventional formulations.

## 2 Materials and Methods

### 2.1 Materials

The following chemicals were kindly supplied by Westlake Epoxy GmbH: the epoxy resin DGEBA (EPIKOTE Resin 04976, DG from then on) with an EEW of 184–190 g eq^–1^, the epoxy mixture of 75–90% DGEBA and 10–20% HDDGE (EPIKOTE Resin MGS RIMR 135; DGHD from then on) with an EEW of 166–185 g eq^–1^, the curing agent tetrahydromethylphthalic anhydride ANHY (EPIKURE Curing Agent 04976) with a molecular weight or rather an anhydride equivalent weight (AEW) of 166.2 g eq^–1^, and the initiator 1-methylimidazole IMI (EPIKURE Catalyst 04976). The monofunctional aromatic epoxy-based reactive diluent 15-M-phenylglycidyl-(*n*-pentadecca-8,11-dienyl) (Cardolite Ultra LITE 513; LI from then on) with an EEW of 350–425 g eq^–1^ was provided by Cardolite Corporation. The following epoxy-based reactive diluents were kindly supplied by Huntsman Corporation: the monofunctional aromatic diluents *p*-tert-butylphenyl 1-(2,3-epoxy)­propyl ether (ARALDITE DY-P; DP from then on) with an EEW of 222–244 g eq^–1^ and 2,3-epoxypropyl-*o*-totyl ether (ARALDITE DY-K; DK from then on) with an EEW of 175–189 g eq^–1^; the difunctional diluent 1,4-bis­(2,3-epoxypropoxy)­butane (ARALDITE DY-D; DD from then on) with an EEW of 118–125 g eq^–1^; and the multifunctional diluent 1,3-propianediol, 2-(hydroxymethyl)-2-methyl-, polymer with (chloromethyl)­oxirane (ARALDITE DY-31; DY from then on) with an EEW of 123 g eq^–1^. The chemical structures of the used chemicals are depicted in Scheme 1. All chemicals were used without any further purification.

**Scheme 1:**
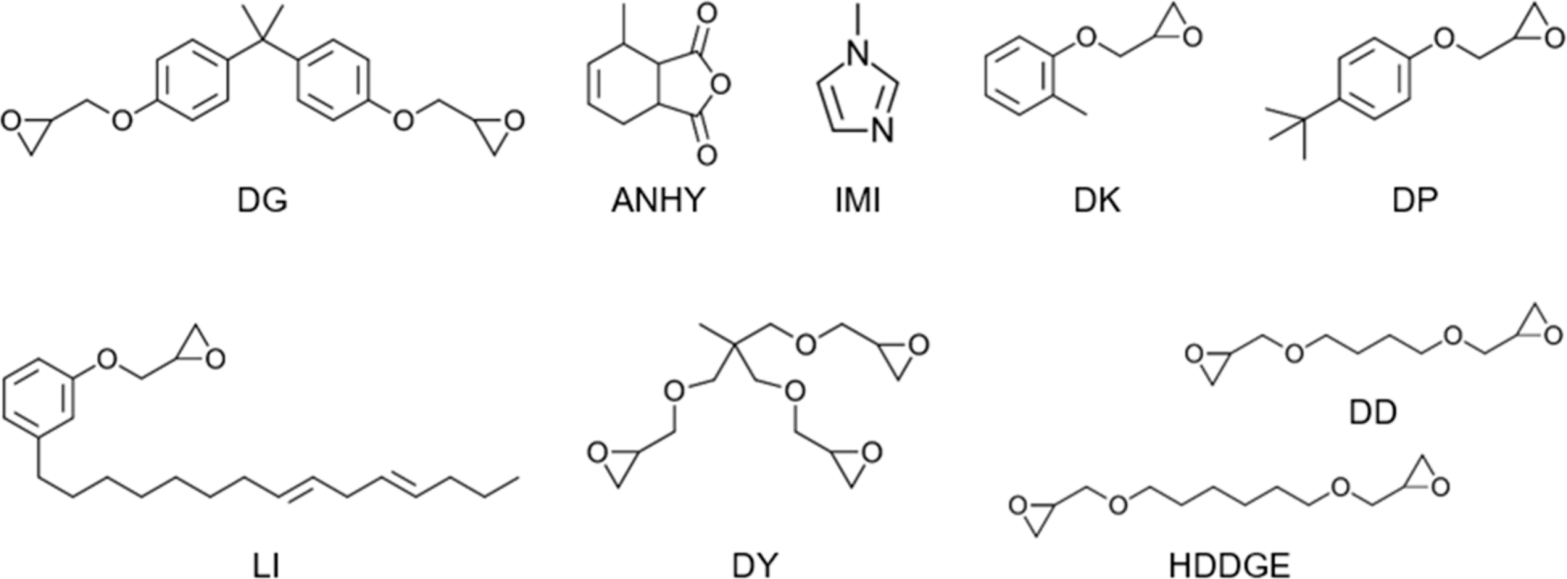
Chemical Structures of all Chemicals Used, as Specified in the Data Sheets of Their Manufactures

### 2.2 Sample Preparation

Most samples were labeled DGx-DILUENTy-ANHYz, where x indicates the amount of DG, y indicates the amount of diluent, and z indicates the percentage content of ANHY in respect to conventional (nearly stoichiometric) ratio between the epoxy mixture DGxDILUENTy and ANHY. The exception here is the commercial epoxy mixture DGHD, for which only estimated amounts of DG and HDDGE are given in the safety data sheet. The parts by weight of anhydride to be used with a hundred parts by weight of epoxy resin were calculated by dividing AEW by EEW of DGxDILUENTy to determine the stoichiometric ratio of ANHY to use with DGxDILUENTy.^41,52^ In the case of conventional anhydride-epoxy thermosets, the nearly stoichiometric ratio is usually considered sufficient to obtain optimum properties,^41,43,52–54^ due to significant epoxy homopolymerization,^41^ which could otherwise leave unreacted anhydride at the end of curing.^54^ Therefore, the value obtained was then multiplied by 0.9 for thermosets containing HDDGE, LI, DK and DP, and by 0.7 for thermosets containing DD and DY. The multiplication factor was reduced for samples containing DD and DY, because otherwise the amount of ANHY would exceed the amount of diluent in the formulations due to their low EEW values. The EEW values of DGx-DILUENTy-ANHYz-IMI1.5 formulations were calculated in the following way^55^

1 $${EEW}_{DGxDILUENTy}=\frac{W_{DG}+W_{DILUENT}}{\left(\frac{W_{DG}}{{EEW}_{DG}}\right)+\left(\frac{W_{DILUENT}}{{EEW}_{DILUENT}}\right)}$$

In eq 1, *W*_DG_ and *W*_DILUENT_ are the weights of DG and diluent, while EEW_DG_ and EEW_DILUENT_ are EEW values of DG and diluent, respectively.^55^ Sample coding and composition of all the formulations is shown in Table 1.

**Table 1:** Sample Coding and Composition of All the Formulations

formulation	DG (g)	DGHD (g)	diluent (g)	ANHY (g)	IMI (g)	EEW (g eq^–1^)
DGHD-ANHY35		10		2.98	0.15	175.5
DG80LI20-ANHY35	8		2	2.51	0.15	208.6
DG70DP30-ANHY35	7		3	2.64	0.15	198.8
DG80DK20-ANHY35	8		2	2.81	0.15	186.0
DG80DD20-ANHY35	8		2	2.91	0.15	168.8
DG60DY40-ANHY35	6		4	3.00	0.15	154.8
DG80DD20-ANHY40	8		2	3.37	0.15	168.8
DG80DD20-ANHY42.5	8		2	3.58	0.15	168.8
DG80DD20-ANHY45	8		2	3.79	0.15	168.8
DG80DD20-ANHY100	8		2	8.42	0.15	168.8

For determination of curing conditions, DG80DD20-ANHYz samples (for 100 g DG each time) were vigorously stirred at RT, and precured at 45 °C between 9 and 14 h in aluminum dishes with a diameter of approximately 13.5 cm. All DG80DD20-ANHYz final materials with *z* = 40, 42.5, and 45 were cured under selected precuring conditions, then at 100 °C for 1 h, and postcured at 150 °C for 7 h (see Table S1). DG80DD20-ANHY100 sample was cured under four different curing conditions: (a) 4 h 80 °C and 2 h 130 °C; (b) 4 h 80 °C and 7 h 150 °C; (c) 4 h 80 °C and 2 h 130 °C and 7 h 150 °C; and (d) 24 h 45 °C and 1 h 100 °C and 7 h 150 °C. For characterization of thermomechanical properties (DMA) and mechanical properties (tensile and 3-point bending testing), the final materials were cured in closed metal molds to obtain the appropriate sample thickness.

### 2.3 Rheology Analysis

Isothermal rheological measurements of DG and DGxDILUENTy (without curing agent and initiator) with different y ranging from 10 to 50 (*x* + *y* = 100 each time, e.g., in the case of DG50DILUENT50, this is 5 g of DG + 5 g of DILUENT = 10 g total sample weight) were recorded at RT on a parallel plate rheometer ARES from Rheometric Scientific in oscillation mode at a strain of 100%, a frequency of 20 Hz and a distance between plates of 0.6 mm for 10 min to determine the viscosity.

### 2.4 Differential Scanning Calorimetry

Dynamic DSC measurements of reactive, intermediate and final materials (approximately 5–10 mg) were carried out with Mettler Toledo DSC3+ and evaluated with a STAR^e^ software. The samples were placed in aluminum pans and measured in the temperature range from −80 °C (or 25 °C) to 250 °C with a heating and cooling rate of 10 °C min^–1^ under a constant nitrogen flow of 50 mL min^–1^. DSC was used to monitor the transition temperatures of un-cross-linked polymer *T*_g0_^DSC^, maximum peak temperatures *T*_p1,r_^DSC^ and *T*_p2,r_^DSC^, enthalpies of two exothermic peaks Δ*H*_1r_^DSC^ and Δ*H*_2r_^DSC^ and an additional exothermic region Δ*H*_3r_^DSC^ (obtained from the first heating run), and glass transition temperatures *T*_g1_^DSC^ (obtained from the second heating run) for the reactive materials. The glass transition temperatures *T*_gi_^DSC^, maximum peak temperatures *T*_p,i_^DSC^, and remaining enthalpies of exothermic peaks Δ*H*_i_^DSC^ were determined for the intermediate materials, and the glass transition temperatures *T*_gf_^DSC^ for the final materials.

### 2.5 Dynamic Mechanical Analysis

Dynamic DMA measurements of final materials were performed on a DMA Q800 from TA Instruments, equipped with a single cantilever clamp. Rectangular specimens, measuring approximately 35 × 10 × 2 mm^3^, were studied at a frequency of 1 Hz, and in a temperature range from 0 to 200 °C with a heating rate of 2 °C min^–1^. The glass transitions of the final materials *T*_gf1_^DMA^ and *T*_gf2_^DMA^ (obtained from the first and second heating runs) were determined as the temperatures at which the maximum in the tan delta curve in the α transition region occurred,^28^ and used to calculate the full width at half-maxima *fwhm*_1_ and *fwhm*_1_ in order to investigate the heterogeneity of the networks.^2,32^ The cross-link densities *v*_ef1_^DMA^ and *v*_ef2_^DMA^ were then calculated as follows^56^

2 $$v_{ef}^{DMA}=\frac{Ǵ}{RT}=\frac{É}{3RT}$$

In eq 2, *E* ^´^ is the storage modulus value in dyn cm^–1^ obtained in the rubbery plateau region at the temperature of 50 K after *T*_gf_^DMA^, *R* is the gas constant equal to 8.314 × 10^7^ dyn cm K^–1^ mol^–1^, and *T* is the temperature corresponding to the *E* ^´^ value.^56^ The changes in network density were further analyzed by subtracting the storage modulus from loss modulus Δ­(*E* ^´^,*E*″) in the rubbery plateau region, as their difference increases with increasing cross-link density.^57^ The molecular weights between cross-links *M*_c1_^DMA^ and *M*_c2_^DMA^ can be then calculated based on the *v*_ef_^DMA^ value and the density ρ ^58–61^

3 $$M_{c}^{DMA}=\frac{\rho }{v_{ef}^{DMA}}$$

The first value of storage modulus obtained at 0 °C, designated as *E* ^´^(0°), was also determined.

### 2.6 Tensile Testing

Tensile testing of final materials was carried out on a universal testing machine Zwick 1445 from ZwickRoell AG. At least 5 samples (type 1B) of each type with a gauge length of 50 mm, width of 10 mm and thickness of 4 mm were measured at a testing speed of 1 mm min^–1^ according to DIN EN ISO 527-2.

### 2.7 Three-Point Bending Flexural Testing

Three-point bending flexural testing of final materials was performed on a universal testing machine Zwick 1445 from ZwickRoell AG. At least five samples of each type, measuring 80 × 10 × 4 mm^3^, were studied at a testing speed of 2 mm min^–1^ according to DIN EN ISO 178.

### 2.8 Thermogravimetric Analysis

TGA measurements of final materials (approximately 10 mg) were carried out with a Mettler Toledo TGA/DSC3+. The samples were placed in aluminum oxide ceramic crucibles and measured in the temperature range from 25 to 800 °C with a heating rate of 10 °C min^–1^ under a constant air flow of 50 mL min^–1^. The temperatures at which the mass reduction of 5 and 50 wt % occurred were determined and labeled *T*_–5wt–%_ and *T*_–50wt–%_, respectively. The first derivative of the TGA curves was further analyzed and its temperature of maximum mass change rate *T*_max_ was determined.

### 2.9 Fourier-Transform Infrared Spectroscopy

FTIR experiments were conducted on reactive materials using a FTIR spectrometer HAAKE MARS 60 Rheometer from Thermo Fischer Scientific GmbH, and on intermediate and final materials using an iS50 from Thermo Scientific, both equipped with an attenuated total reflection (ATR) accessory. FTIR spectra were recorded at RT in the wavelength range from 4000 to 400 cm^–1^ with a resolution of 4 cm^–1^ averaging 32 scans for each spectrum to monitor the changes in functional groups at different stages of the curing process. The spectra were normalized using the peak at 1508 cm^–1^ associated with aromatic rings of DG.^62,63^

## 3 Results and Discussion

### 3.1 Preliminary Results

First of all, we investigated the possibility of using various diluents to create sequential dual-curing epoxy-anhydride thermosets with low viscosity and high *T*_g_ values, which do not cause postcuring difficulties, as observed in the case of thermosets containing HDDGE in our previous study. At RT, the viscosity of various DGxDILUENTy samples (without curative to eliminate reactivity effects) was determined; for these samples, the y values ranged from 10 to 50, i.e., from 1 to 5 g diluent per total sample mass of 10 g (epoxy resin with diluent). The viscosity values were then plotted against the amount of the diluent to find the epoxy mixture with a viscosity closest to DGHD, which is approximately 1.3 Pa × s (see Figure 1).

**Figure 1:**
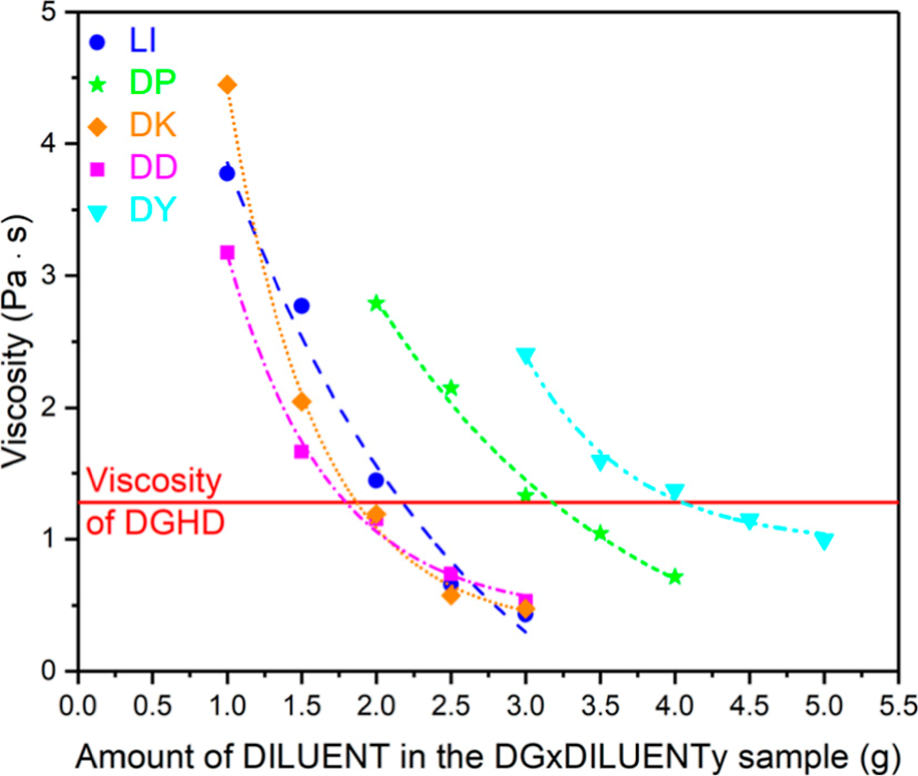
Viscosity as a function of the amout of the diluent at RT (the total mass of the sample is 10 g).

Next, DGxDILUENTy samples with the desired viscosity closest to DGHD were chosen to prepare DGxDILUENTy-ANHY35 formulations, which were dynamically cured at 10 °C min^–1^ using DSC (see Figure 2a). Each DSC curve, regardless of the diluent used, exhibits a *T*_g0_^DSC^ of −40.4 ± 2.7 °C, which is probably due to the similar initial viscosity of formulations, and two exothermic peaks, confirming their dual curing behavior. The first curing peak indicates base-catalyzed anhydride-epoxy condensation^30^ or esterification,^50,51^ while the second curing peak indicates epoxy homopolymerization^30^ or etherification,^50,51^ respectively. Both exothermic peaks tend to shift toward lower temperatures with increasing functionality of a diluent and decreasing EEW. Thus, the DG60DY40-ANHY35 and DG80DD20-ANHY35 formulations containing a multi- and difunctional diluent exhibit the highest reactivity with the lowest maximum peak temperatures and the highest enthalpies of exothermic regions (the exact values are given in Table 2). The DG60DY40-ANHY35 formulation owes its superior reactivity, in addition to its superior functionality, to the lack of long aliphatic chains and aromatic rings that could cause steric hindrance. In turn, the formulations containing monofunctional aromatic diluents such as DK, DP and LI exhibit lower reactivity with higher maximum peak temperatures and lower enthalpies of exothermic regions (see Table 2).

**Figure 2:**
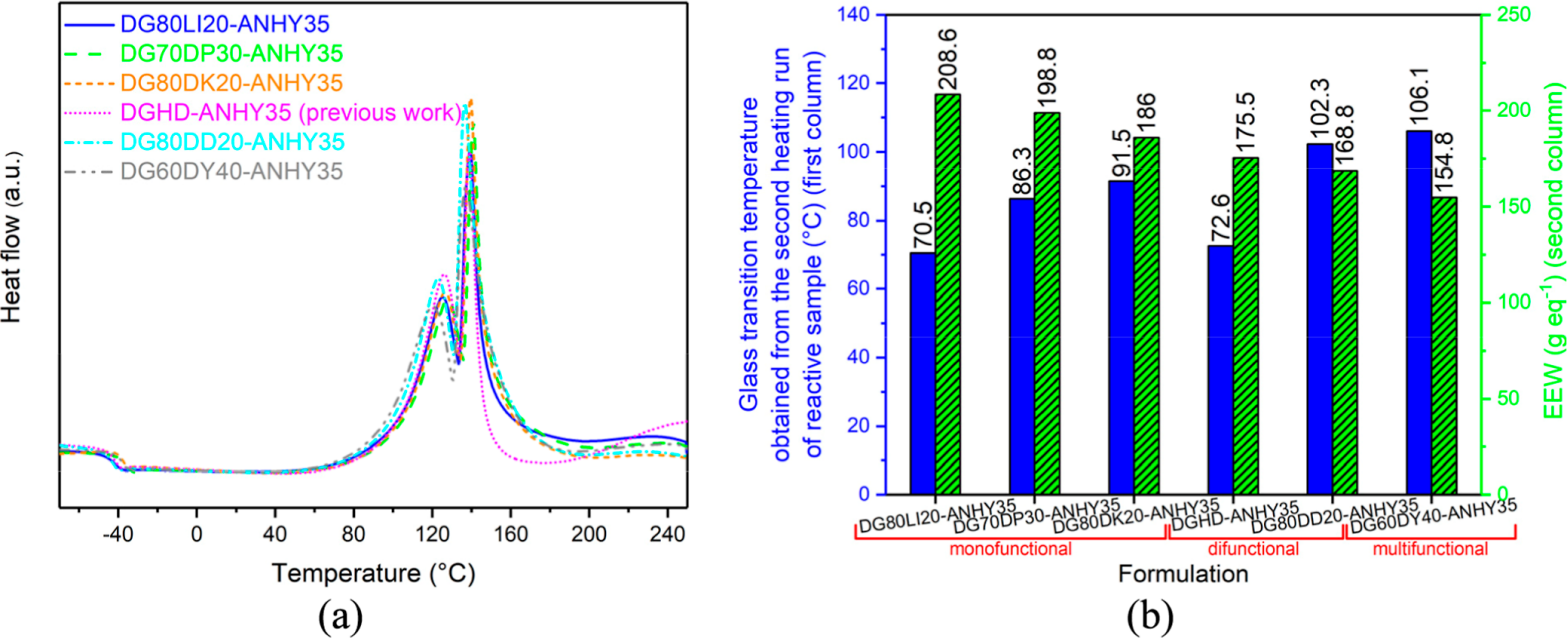
DSC curves of reactive DGxDILUENTy-ANHY35 samples (a), and glass transition temperatures of DGxDILUENTy-ANHY35 samples in relation to their functionality and EEW values (b). The result of the DGHD-ANHY35 sample from our previous study, which was coded there as DG-ANHY35-IMI1.5, is also shown.^40^

**Table 2:** DSC Data of Reactive Materials

formulation	EEW (g eq^–1^)	*T*_g0_^DSC^ (°C)	*T*_p1,r_^DSC^ (°C)	*T*_p2,r_^DSC^ (°C)	Δ*H*_1r_^DSC^ (J g^–1^)	Δ*H*_2r_^DSC^ (J g^–1^)	Δ*H*_3r_^DSC^ (J g^–1^)	*T*_g1_^DSC^ (°C)
DG80LI20-ANHY35	208.6	–42.4	125.5	139.3	123.9	125.8	4.3	70.5
DG70DP30-ANHY35	198.8	–37.6	127.9	140.9	126.3	136.2	1.5	86.3
DG80DK20-ANHY35	186.0	–38.3	126.4	139.7	138.4	152.4	1.8	91.5
DGHD-ANHY35	175.5	–42.1	126.2	139.1	152.9	89.1	47.8	72.6
DG80DD20-ANHY35	168.8	–43.9	122.9	136.5	144.8	189.2	3.2	102.3
DG60DY40-ANHY35	154.8	–38.4	120.5	137.3	139.2	164.3	3.2	106.1

In addition to the two expected exothermic peaks indicating two competing reactions (esterification, etherification), an additional exothermic region above approximately 176 °C has been observed for the DGHD-ANHY35 sample in our previous study (see Figure 2a). This region was interpreted as an additional reaction related to a small amount of HDDGE that is most likely the etherification of hydroxyl groups coming from HDDGE. This region represents 16.5% of the total enthalpy (including this region), which was found to cause undesired postcuring difficulties, resulting in low *T*_g_ values of the final material.^40^ It was therefore crucial to investigate whether other diluents also exhibit significant exothermic regions at high temperatures, that is, in addition to those expected for the esterification and etherification. And indeed, replacing HDDGE with any another diluent investigated in this study significantly reduces the contribution of this region to total enthalpy to only 0.6–1.7% (see Table 2). This suggests that this phenomenon should eliminate the need to use final curing temperatures higher than 150 °C in order to achieve relatively stable *T*_g_ values, which will be examined in detail later.

*T*_g1_^DSC^, like reactivity, generally increases with increasing functionality of a diluent and decreasing EEW of the formulations (see Figure 2b), with the sole exception of DGHD-ANHY35. This correlates with the structure of the individual diluents. The multifunctionality combined with the lack of long aliphatic chains and aromatic rings results in DG60DY40-ANHY35 having the highest *T*_g1_^DSC^. DG80DD20-ANHY35 ranks second due to the difunctionality combined with the presence of a flexible, straight four-carbone (butane) chain between the two epoxy groups. DG80DK20-ANHY35 places third due to the monofunctionality and presence of one aromatic ring. DG70DP30-ANHY35 ranks fourth due to the same functionality and similar structure to DK but a higher content of the diluent used. Finally, DG80LI20-ANHY35 ranks last due to undesired characteristics of LI such as monofunctionality, one aromatic ring and a very long aliphatic chain. DGHD-ANHY35 deviates from the expected trend; based on its EEW, it should rank third. The most likely explanation for the discrepancy between prediction and observation is the additional exothermic region Δ*H*_3r_^DSC^ caused by the two additional methyl groups in the backbone compared to DD, which does not provide clear information on *T*_g1_^DSC^. Considering all available information on reactivity and *T*_g_ values, DD was chosen as diluent for further investigation. DG60DY40-ANHY35 was rejected despite its highest *T*_g_ due to its significantly higher reactivity compared to DGHD-ANHY35 sample, and due to the possible negative impact of the high content of DY in the formulation on other important properties of the final thermoset. Further analysis also showed that significant changes in viscosity were observed for DG80DD20-ANHYz with z ranging from 40 to 45, and these formulations will be presented below.

### 3.2 Curing Behavior

At the beginning of this study, four DSC measurements of reactive DG80DD20-ANHYz samples were performed to investigate changes in the relative contribution of the two curing reactions (esterification and etherification) in dependence on the amount of ANHY in the formulation (see Figure 3 and Table S2). *T*_go_^DSC^ decreases with increasing amount of ANHY due to decreasing viscosity as well as decreasing amount of DG80DD20 relative to the amount of ANHY. An increase in the amount of ANHY in the formulation increases the contribution of esterication in relation to etherification, which causes an increase in enthalpy of the first exothermic peak and a decrease in the enthalpy of the second exothermic peak. This is consistent with our previous investigation on DGHD-ANHYz systems (there were samples labeled DG-ANHYx-IMI1.5).^40^ The results indicate that the maximum peak temperatures shift toward higher temperatures, and this delay in the initiation of the reaction by nucleophilic attack of IMI to the epoxy ring with increasing amount of ANHY in the formulation is a phenomenon commonly observed in other similar epoxy-anhydride thermosets.^30^ Furthermore, in the case of the three off-stoichiometric samples, the maxima of the two curing peaks differ by only 12.9 ± 0.2 °C, resulting in a large overlap, similar to that observed in HDDGE-containing samples,^40^ which in turn prevents the two reactions from proceeding in a fully sequential manner. To confirm this, these samples (100 g DG) were precured at 45 °C in aluminum dishes for different times and measured by DSC (see Figure 3b–d and Table S3).

**Figure 3:**
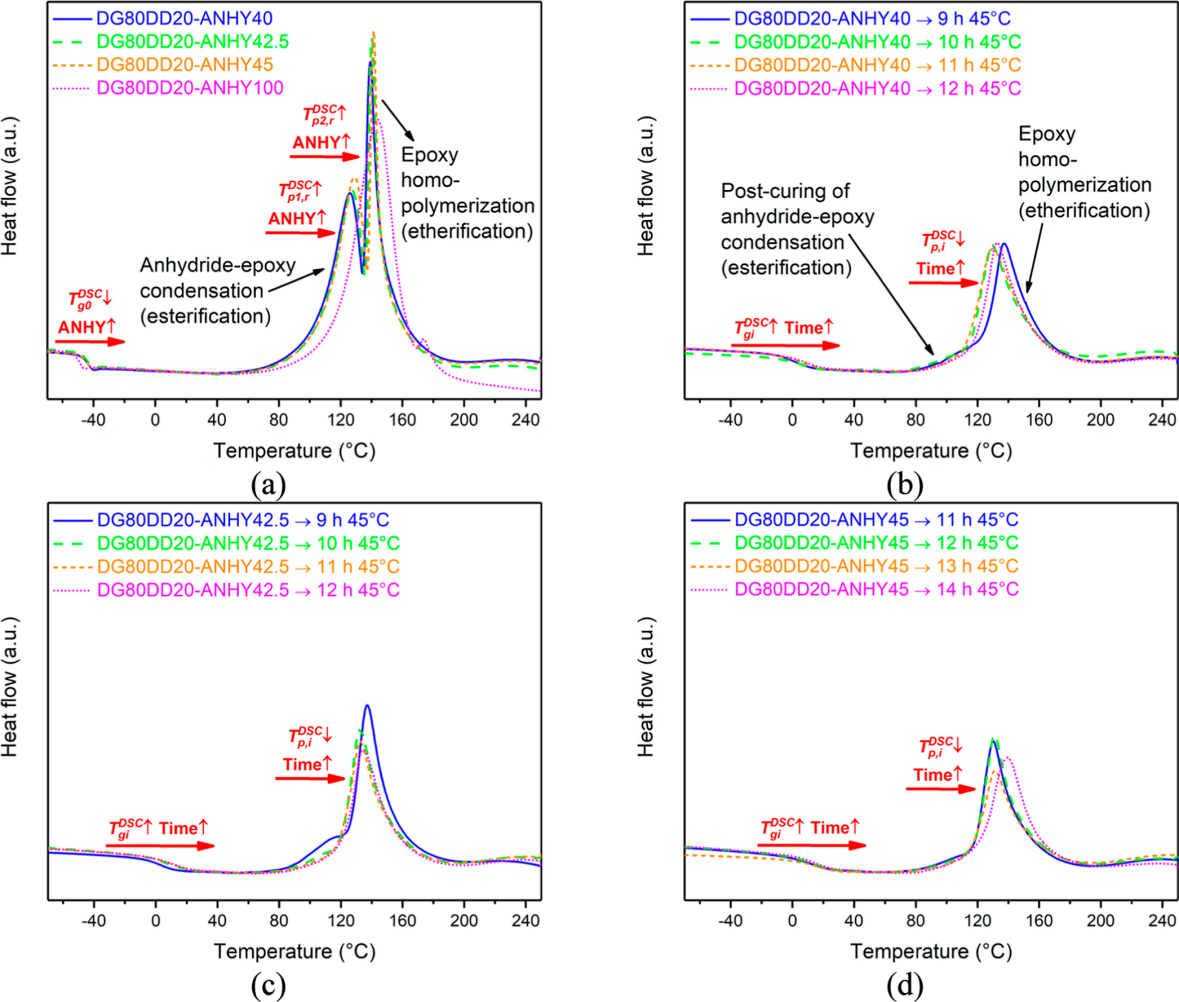
DSC curves of reactive DG80DD20-ANHYz samples (a), and of intermediate DG80DD20-ANHYz samples (b–d).

The quasi-sequentiality can indeed be observed from the continuous increase in *T*_gi_^DSC^, and the decrease (or in some cases very similar values) in the Δ*H*_i_^DSC^ and *T*_p,i_^DSC^ values of intermediate DG80DD20-ANHYz materials with increasing precuring times. Furthermore, the shoulder indicating postcuring of the first curing stage or ending of anhydride-epoxy condensation^30^ disappears over the precuring time. The low *T*_gi_^DSC^ values (significanlty lower than the chosen precuring temperature),^64^ as well as a general shift of *T*_p,i_^DSC^ values toward lower temperatures with increasing precuring time, indicate that intermediate materials have not vitrified so far.^51^ Therefore, the intermediate materials cannot be stored indefinitely at RT, although further curing can still be impeded for some time. The overlapping of the two curing stages is also confimed by the continuous changes in viscosity during the activation of the second curing stage observed with increasing precuring time (see Figure S1). As more active groups react during precuring, fewer are available for subsequent reaction, resulting in a smaller increase in viscosity with increasing precuring time. The decrease in viscosity depends on both the precuring time and the amount of ANHY in the formulations, and this change occurs within a relatively narrow range (precuring time of between 10 and 12 h). The light yellow intermediate materials change color to dark violet during the activation of the second curing stage, which is a sign of the formation of initiator fragments with conjugated double bonds attached to one of the chain ends, i.e., a sign of activated anionic epoxy homopolymerization.^65^ The observed changes are consistent with those described in our previous study, except for the individual values (the curing process is faster, the *T*_gi_^DSC^ values are higher this time, etc.).^40^ A slight shoulder in the DSC curves can be still observed for DG80DD20-ANHY40 and DG80DD20-ANHY42.5 intermediate materials after 10 h of precuring at 45 °C, and for DG80DD20-ANHY45 after 12 h, and these precuring conditions were chosen for further analysis.

The intermediate materials were further cured at 100 °C for 1 h, followed by postcuring at 150 °C for 7 h, and measured again using DSC (see Table S4). The off-stoichiometric samples show *T*_gf_^DSC^ values of approximately 107.7 ± 2.5 °C after the curing at 100 °C, and after further postcuring at 150 °C, they shift toward 135.1 ± 1.0 °C. Although the shift is still large after postcuring, the minor contribution of the additional exothermic enthalpy occurring after the two exothermic peaks observed in the DSC of the reactive samples allows to achieve significantly higher *T*_g_^DSC^ values after the activation of the second curing stage at 100 °C compared to off-stoichiometric thermosets containing HDDGE investigated in our previous work (with the maximum value of 52.3 °C).^40^ In the next section, DMA will be used to examine whether these curing conditions are optimal for obtaining relatively stable *T*_g_ values, or whether these values will continue to increase.

### 3.3 Thermomechanical Properties

The evolution of tan delta and storage modulus of DG80DD20-ANHYz final materials, determined using DMA, is shown in Figure 4a, and the corresponding data are collected in Table S5. Each sample was measured twice.

**Figure 4:**
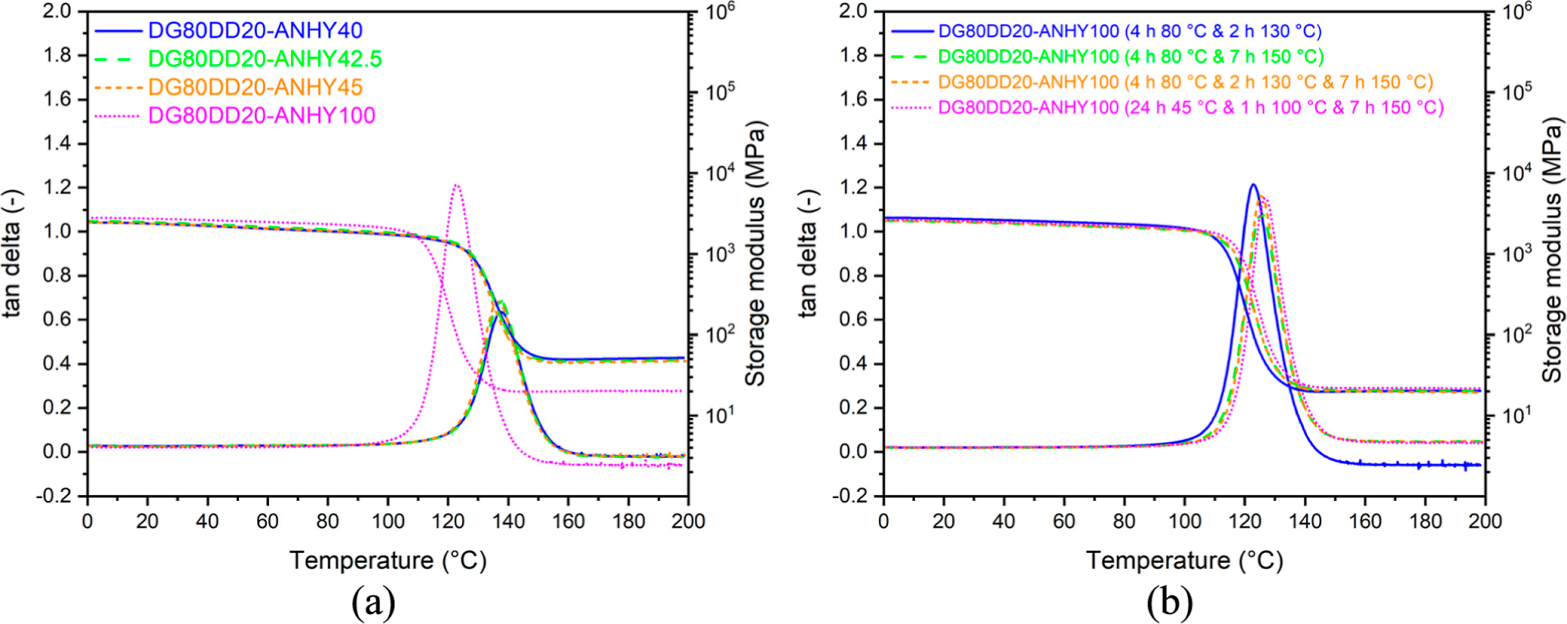
DMA curves of DG80DD20-ANHYz final samples (a), and of DG80DD20-ANHY100 samples after different curing conditions (b).

All final materials show unimodal peaks without shoulders, which indicates high homogeneity and miscibility. Their homogeneity is also very similar, as the *fwhm*_1_ values are approximately 15.5 ± 0.4 °C, regardless of the relative contribution of both curing stages. The conventional (nearly stoichiometric) DG80DD20-ANHY100 formulation is expected to exhibit a narrower relaxation than the other formulations due to the dominance of the condensation reaction,^28^ which is indeed slightly higher when the same samples are measured once again (see *fwhm*_2_ values in Table S5). Another characteristic feature of the dominant condensation reaction in the formulation described in the literature is the high tan δ peak.^28^ In fact, the conventional sample exhibits a significantly higher tan δ peak than the off-stoichiometric formulations, in this case is almost twice as high. The relaxation curves of the off-stoichiometric samples are shifted toward higher temperatures compared to the conventional sample. This leads to higher *T*_gf1_^DMA^ values of the off-stoichiometric samples of approximately 137.1 ± 0.5 °C compared to the *T*_gf1_^DMA^ value of the conventional sample of 122.9 °C. This observation, although not confirmed in a similar study on dual-curable off-stoichiometric epoxy-anhydride thermosets,^30^ is commonly reported for dual-curable off-stoichiometric epoxy-thiol^1,2^ and epoxy-amine thermosets.^28^ Consequently, the off-stoichiometric samples show *v*_ef1_^DMA^ values of 0.00427 ± 0.00020 mol cm^–1^ and ${\Delta \left(É,É{´}^{´}\right)}_{1}$ values of 50.0 ± 2.4 MPa, while the conventional sample shows *v*_ef1_^DMA^ value of 0.00182 mol cm^–1^ and ${\Delta \left(É,É{´}^{´}\right)}_{1}$ value of 21.5 MPa.

The difference in *T*_gf_^DMA^ between the first and second DMA measurement of all off-stoichiometric formulations is far below 1.2 °C, indicating that further exposure to heat does not cause further postcuring. This difference, however, is slightly higher for the conventional sample and amounts to 2.7 °C. It is worth noting that the *T*_gf1_^DMA^ and *T*_gf2_^DMA^ values of the conventional sample are also very similar to those of the conventional sample containing HDDGE determined in our previous study, which are 124.2 and 126.3 °C.^40^ There is no increase in *T*_g_ caused by the change of a diluent for the conventional sample, as observed for off-stoichiometric samples. The *T*_g_ values of the final materials depends strongly on the choice of diluent, but only at high contribution of epoxy homopolymerization. The DG80DD20-ANHY100 formulation was cured under different curing conditions, i.e., the final curing temperature of 130 °C was lower, and the curing time of 2 h shorter. For better comparison, three alternative curing cycles were chosen to deepen this analysis, all with the final curing step at 150 °C for 7 h (see Figure 4b, Table S6). All alternative curing cycles resulted in higher *T*_gf1_^DMA^ values, with the highest value of 126.7 °C achieved after the longest curing cycle. The *T*_gf1_^DMA^ is still lower than that of the off-stoichiometric samples.

Higher *T*_gf_^DMA^ values of off-stoichiometric thermosets indicate limited free volume of the chain segments and reduced ability to move in various directions due to the increased number of chemical cross-links, resulting in significantly lower *M*_c_^DMA^ values.^61,66^ Typically, such reduced mobility in either side chains or small groups of adjacent backbone atoms leads to lower compliance of the molecule and higher storage modulus in the glass region;^61^ however, this is not the case here. The ${É\left(0^{{}^{\circ}}\right)}_{1}$ value of off-stoichiometric samples is 2.4 ± 0.1 GPa, which is lower than ${É\left(0^{{}^{\circ}}\right)}_{1}$ value of the conventional sample of 2.8 GPa (see Table S5), and also lower than the value of DGHD-ANHY35 (previously coded as DG-ANHY35-IMI1.5) of 3.0 GPa (previously not even considered and discussed). The increase in glassy storage modulus indicates better packing of all chain fragments,^61^ which appears to be result from the chemical cross-links introduced by the anhydride curing agent in the conventional sample, and which consequently leads to a significantly higher contribution of esterification. Several explanations for this phenomenon can be considered. First, the drastic decrease in the glassy storage modulus may result from a higher amount of reactive diluent (DD) in off-stoichiometric formulations, which is consistent with the observation described in the literature that the glassy storage modulus decreases sharply with increasing amount of diluent.^55,67^ However, the changes are usually accompanied either by a simultaneous decrease in *T*_gf_^DMA^,^67^ caused by increased mobility resulting from the introduction of aliphatic chains in the structure^68^ (plasticizing effect^67^) (which is not observed in this study) or by a broadening of the tan δ peak due to a higher degree of microstructural heterogeneities^55^ (only minor changes are observed in this study). The explanation based on the presence of the diluent does not seem convincing in this case, and the reason may be that the difference in the amount of DD is too small (10.8% compared to 14.6%). Second, the increase in the glassy storage modulus may indicate antiplasticization and is typically accompanied by a decrease in *T*_gf_^DMA^.^69–72^ Nevertheless, this phenomenon is either enhanced by increasing the amount of diluent,^69,70^ or occurs in off-stoichiometric epoxy systems,^71,72^ neither of which is observed in this case. Third, the drop in the storage modulus combined with restricted mobility in off-stoichiometric samples can be related to increased sensitivity toward the applied stress during the DMA measurement, which, in turn, can be observed by comparing *T*_gf1_^DMA^ and *T*_gf1_^DSC^ values.^73^ The *T*_gf1_^DMA^ values of off-stoichiometric formulations are only slightly higher than *T*_gf1_^DMA^ values (less than 4 °C), whereas this difference is 4.3 °C for the conventional formulation, indicating lower molecular packing^73^ in the off-stoichiometric formulations. For comparison, *T*_gf1_^DMA^ of thermosets containing HDDGE from our previous study, regardless of stoichiometry, are approximately 7.2 ± 1.4 °C higher than *T*_gf1_^DSC^.^40^ This deterioration in ${É\left(0^{{}^{\circ}}\right)}_{1}$ values of off-stoichiometric DG80DD20-ANHYx formulations suggest significant changes in mechanical properties compared to conventional formulations. To dispel any doubts, mechanical properties were determined in the next step.

### 3.4 Mechanical Properties

The mechanical properties of final materials were determined by tensile and three-point flexural tests (see Figure 5).

**Figure 5:**
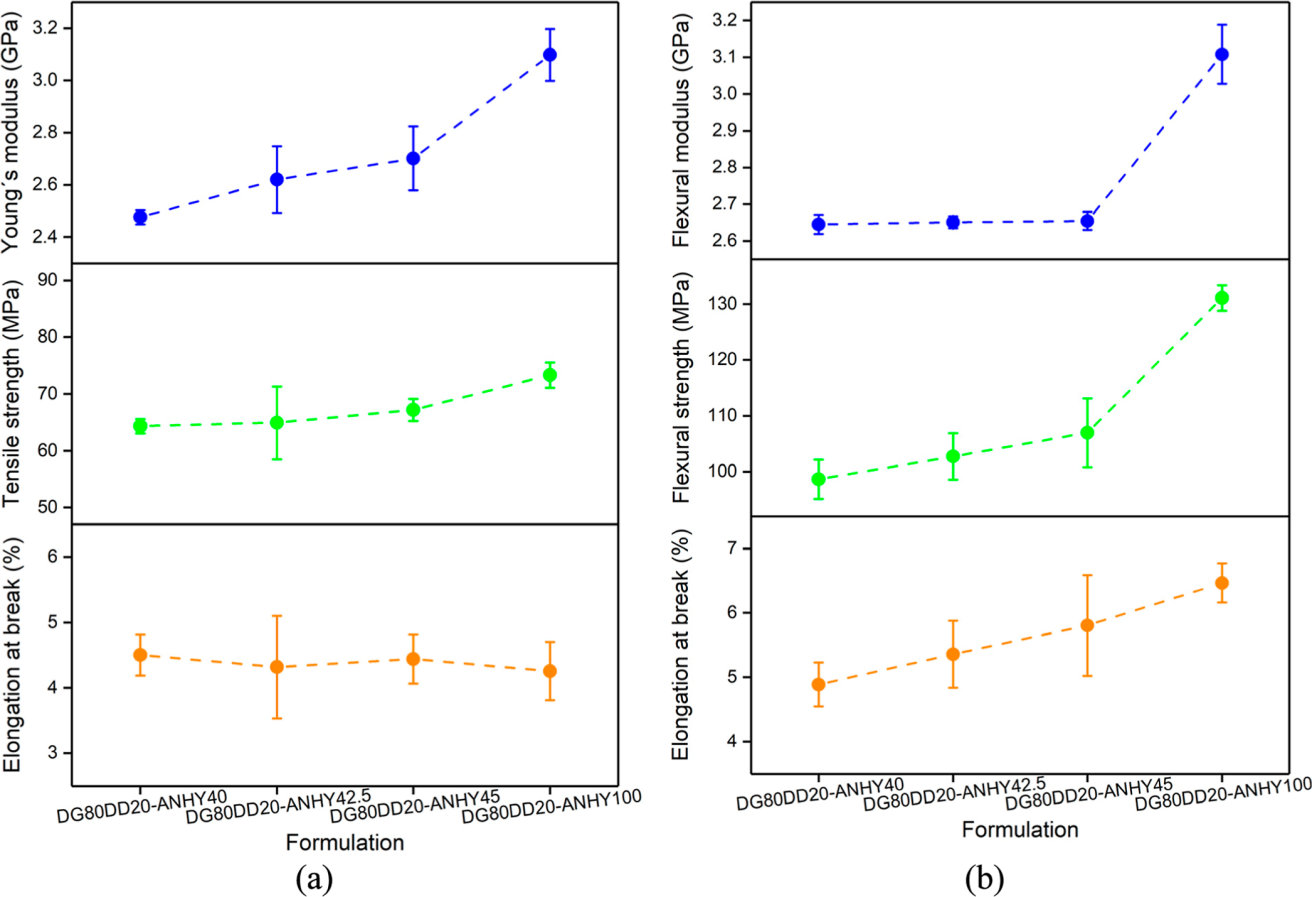
Tensile (a), and flexural (b) properties of DG80DD20-ANHY100 final samples.

The average mechanical (tensile as well as flexural) modulus of the off-stoichiometric samples is 2.6 ± 0.1 GPa, which is significantly lower than the mechanical modulus of the conventional sample, which is 3.1 GPa, confirming the lower modulus observed in the DMA measurement. The decrease in mechanical modulus is accompanied by a corresponding reduction in mechanical strength. The off-stoichiometric samples show tensile strength of 65.5 ± 1.5 MPa and flexural strength of 102.8 ± 4.2 MPa, while the conventional sample shows tensile strength of 73.3 MPa and flexural strength of 131.1 MPa. The conventional interpretation would be to attribute the reduced strength and stiffness to a hypothetical plasticizing effect caused by the reactive diluent (DD) in the off-stoichiometric thermosets. However, if this were the case, one would expect higher elongation at break;^55^ which in this case is comparable to the value for the conventional sample, or even lower than it (tensile elongation of 4.4 compared to 4.3%, and flexural elongation of 5.3 compared to 6.5%). It should therefore be noted that DD, due to its short aliphatic backbone, restricts molecular movements and forms a compact and highly cross-linked structure (as shown in previous section), which significantly reduces the material flexibility. This increased brittleness of the resulting material leads to greater sensitivity to applied stress, resulting in a drop in all mechanical properties of off-stoichiometric formulations. A similar observation was noted in the case of copolyimides, where this phenomenon was explained by lower molecular packing.^73^ In another study, it was indeed found that the poor packing of the molecules in the highly cross-linked samples arises from constraints on the packing of molecules imposed by cross-links. This leads to greater free volume in the glassy state and at the *T*_g_, which might result in lower stiffness.^74^ This observation prompts a suggestion that poor molecular packing is strictly related to the etherification reaction, which is not able to form effective cross-links as readily as the esterification. Consequently, the off-stoichiometric samples contain fewer effective cross-links due to the lower contribution of esterification reaction caused by the lower amount of anhydride in the formulation, which results in a deterioration in mechanical performance.

### 3.5 Thermal Stability

The thermogravimetric curves of all final materials are shown in Figure 6. It can be observed that the degradative curves of off-stoichiometric samples are shifted toward higher temperatures compared to the conventional sample. Both *T*_–5wt–%_ and *T*_–50wt–%_ values of off-stoichiometric samples are on average 13.5 °C higher than those of the conventional samples, which is related to lower number of ester linkages^75^ (see Table S7).

**Figure 6:**
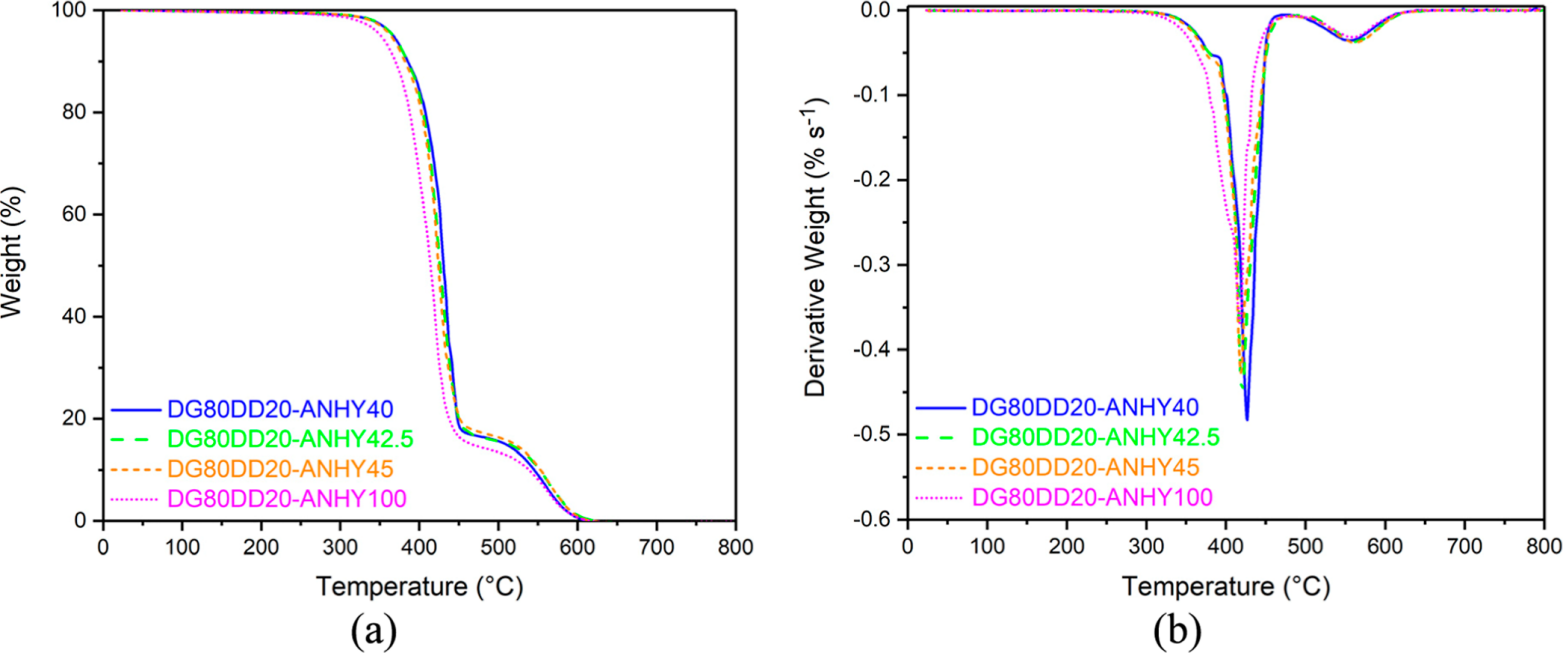
TGA curves (a), and their smoothed derivative curves (b) of DG80DD20-ANHY100-IMI1.5 final samples.

The final materials degrade essentially in two stages, which are considered to be fragmentation and decomposition of the epoxy resin into low molecular weight products in the first stage, leading to the formation of carbonaceous residue, followed by oxidation of this residue at higher temperatures in the second stage.^76,77^ In Figure 6a, the first degradation stage corresponds to the first large negative peak with the *T*_max_ values ranging from 417.9 to 426.5 °C (with decreasing amount of ANHY), and the second one corresponds to the negative peak at approximately 560.2 °C, respectively. In the case of off-stoichiometric samples, the large negative peak has a small shoulder at 376 °C that is attributed to the degradation of labile C–N bonds derived from the structure of IMI.^78^ The second small shoulder at approximately 439 °C, observed in our previous study for off-stoichiometric samples containing HDDGE, which indicated degradation of dual formulations (i.e., faster degradation of ester linkages than ether linkages), does not occur in this case. The most likely cause of this phenomenon is the overlap of these degradation stages due to the higher *T*_max_ of off-stoichiometric samples containing DD compared to off-stoichiometric samples containing HDDGE. For instance, the *T*_max_ for DG80DD20-ANHY35-IMI1.5 is 426.5 °C, while for DGHD-ANHY35 (previously coded as DG-ANHY35-IMI1.5) it is 411.4 °C.

### 3.6 Curing Monitoring

The changes in the functional groups were monitored using FTIR at various curing stages of DG80DD20-ANHYx formulations in order to identify the main curing reactions, which are esterification and etherification^50,51^ (see Figures S2–S4). Before discussing the FTIR results, we attempted to formulate and sketch a possible reaction mechanism for catalyzed diluted anhydride-epoxy thermosets based on the current state of the art, assuming that the course of reaction is the same as for DG due to the presence of epoxy groups in the reactive diluent (see Scheme 2). Initially, the imidazole initiates the curing reaction by opening the epoxy,^50,51,79,80^ or the anhydride ring,^41,43,80^ which is a subject of debate among researchers. If the epoxy ring opens (first possibility in Scheme 2), a zwitterion containing an alkoxide anion is formed, which rapidly opens the anhydride ring to form a species a carboxylic anion.^81^ However, if the anhydride ring opens first, a zwitterion containing a carboxylate anion is formed directly (second possibility in Scheme 2).^41,43^ Regardless of how it is formed, the carboxylic anion reacts with an epoxy group to generate an alkoxide, which in turn reacts with another anhydride to form a carboxylate anion that reacts with another epoxy group;^41,43,64,81,82^ this is how the esterification reaction proceeds, resulting in the formation of polyester-type linkages (highlighted in red).^41,43^

**Scheme 2:**
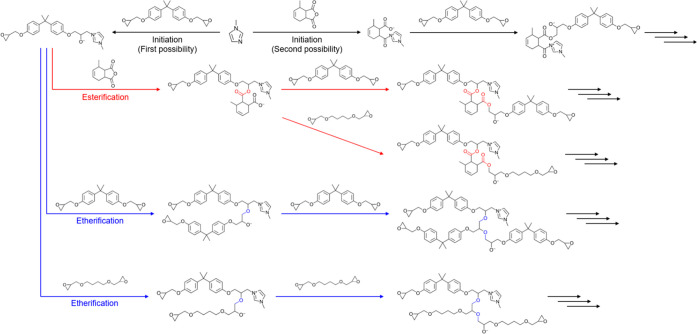
Proposed Reaction Mechanism for Catalyzed Diluted Anhydride-Epoxy Thermosets

The alkoxide anion can also react with the epoxy groups to form ether linkages (highlighted in blue); this reaction is called etherification and occurs competitively with esterification.^50^ Since the reactive diluent contains epoxy groups, both esterification and etherification can proceed in a similar way, as shown in Scheme 2. The main objective of creating sequential dual-curable off-stoichiometric themosets with a deficient amount of a curing agent is to separate the two curing reactions so that they do not occur at the same time and/or at the same temperature. Therefore, in an ideal scenario, during the precuring step at 45 °C, only ester linkages would form, consuming all the reactive anhydride groups and leaving a certain portion of unreactive epoxy groups of DG and DD. These remaining epoxy groups would then undergo homopolymerization at 100 °C, forming ether linkages, as shown in Scheme 3. In reality, this cannot be achieved due to the overlap of the two curing reactions visible in the DSC curves (see Figure 2a), and due to the lack of vitrification after precuring (see Figure 3b–d). There is a certain period of time, during which the two curing reactions compete with each other. Given the unfavorable, nonideal sequential nature of the thermosets under study, FTIR analysis was conducted.

**Scheme 3:**
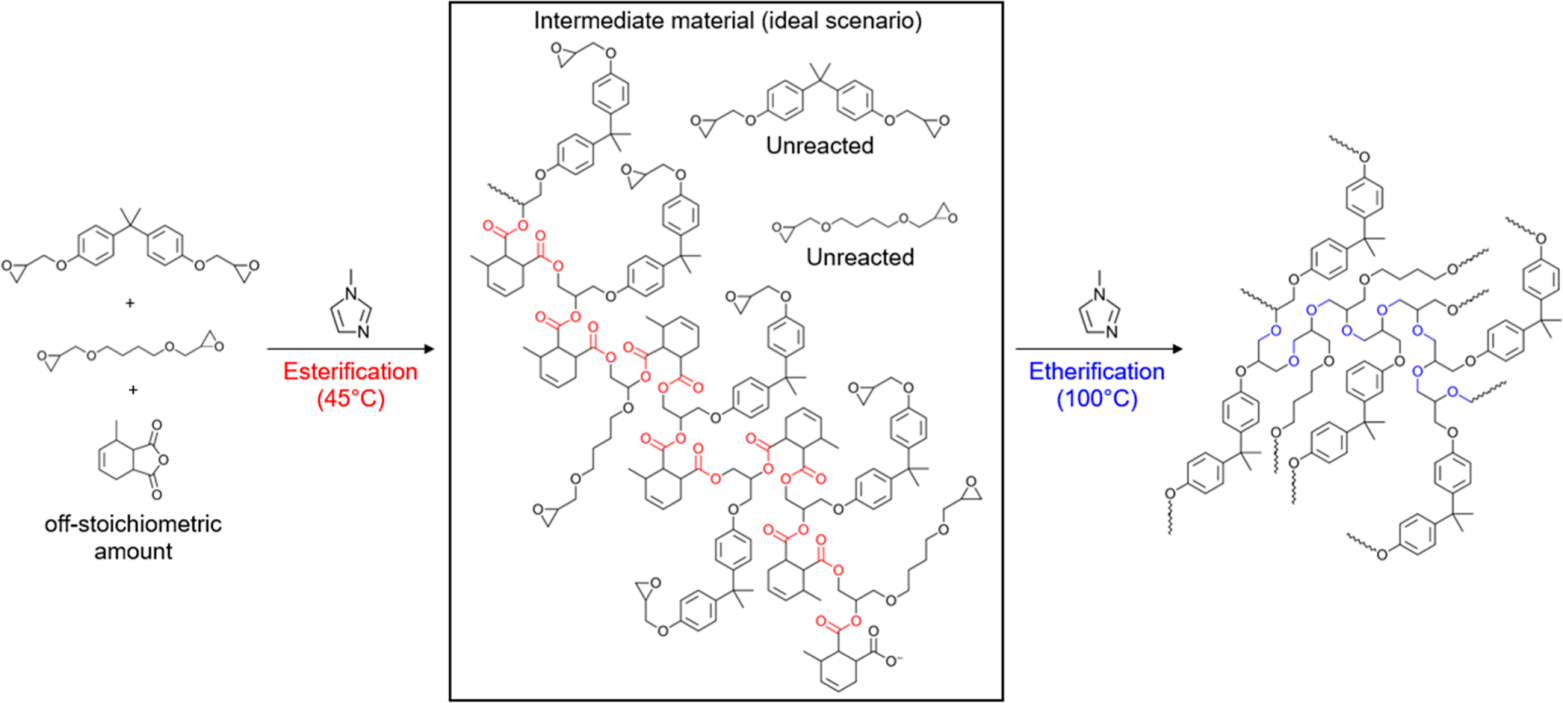
Reaction Scheme Illustrating the Ideal Course of Both Curing Reactions as Well as the Ideal Network Structure of Intermediate Off-Stoichiometric Thermosets

Initially, all reactive specimens possess a characteristic combined epoxy-anhydride band at 914 and 906 cm^–1^,^63,83^ as well as two anhydride bands at 1857 and 1776 cm^–1^.^62,63,78,84–87^ During the first curing stage at 45 °C, the reaction between epoxy and anhydride (esterification) produces ester linkages,^88^ which is why intermediate off-stoichiometric materials do not have any anhydride bands at 906, 1857, and 1776 cm^–1^, only a small epoxy band at 914 cm^–1^, a new ester band at 1732 cm^–1^,^62,84^ and a new broad hydroxyl band at 3489 cm^–1^.^84,89–91^ The isosbestic point, which is the intersection point of the spectral curves at different curing stages, is visible at 1757 cm^–1^ and confirms the successful transformation of anhydride into ester groups.^78^

During the second curing stage at 100 °C, the self-polymerization of epoxy (etherification) produces ether linkages.^88^ The epoxy band at 914 cm^–1^ almost disappears, but the ester band at 1732 cm^–1^ continues to increase slightly, and the hydroxyl band at 3489 cm^–1^ also continues to increase strongly. After the postcuring at 150 °C, final materials do not contain epoxy bands at 914 cm^–1^, but still have hydroxyl bands at 3489 cm^–1^, indicating incomplete etherification, as hydroxyl groups are expected to change into ether groups.^90^ However, researchers report the presence of hydroxyl groups in final materials in some cases.^89,90^ The absence of a distinct ether peak, one that is neither too small^78^ to be detected, nor overlaps with the ester peak,^92^ makes it difficult to determine with absolute certainty at what point the etherification reaction actually begins.

## 4 Summary and Conclusions

The aim of this study was to develop low-reactive and low-viscosity sequential dual-curable off-stoichiometric diluted anhydride-epoxy thermosets characterized by high and stable glass transition temperatures without the need for postcuring temperatures higher than 150 °C. It has recently been observed that an inappropriate choice of diluent can unexpectedly and adversaly lower glass transition temperatures and prolong the curing cycle of off-stoichiometric samples; the selection of 1,6-bis­(2,3-epoxypropoxy)­hexane most likely causes the etherification of hydroxyl groups coming from this diluent to occur at higher temperatures, resulting in the appearance of an additional exothermic region above 176 °C on the DSC curves.^40^ By examining diluents with different chemical structures and functionalities, we concluded that the etherification of hydroxyl groups coming from epoxy resin and diluent can occur within a similar temperature range, resulting in very low contribution of this undesirable exothermic region (less than 1.8%). The glass transition temperature of off-stoichiometric formulations, in turn, generally increases with increasing functionality of the diluent and is the highest when there are no long aliphatic chains and aromatic rings. Therefore, the off-stoichiometric anhydride-epoxy thermosets containing 1,4-bis­(2,3-epoxypropoxy)­butane show high glass transition temperatures of 137.1 °C, determined by DMA technique, which remain constant during the second heating run. These values represent an improvement of approximately 48 °C compared to off-stoichiometric thermosets containing 1,6-bis­(2,3-epoxypropoxy)­hexane postcured under exactly the same conditions. In addition, the developed off-stoichiometric samples exhibit glass transitions 14.2 °C higher than the conventional (nearly stoichiometric) sample, which is also observed in similar off-stoichiometric thiol-epoxy,^1,2^ and amine–epoxy thermosets.^28^ As with the glass transition temperature, the thermal stability of the developed off-stoichiometric formulations is higher than that of both conventional and off-stoichiometric thermosets containing 1,6-bis­(2,3-epoxypropoxy)­hexane.^40^ Despite the desired improvement in the above-mentioned properties, off-stoichiometric thermosets diluted with 1,4-bis­(2,3-epoxypropoxy)­butane have one significant disadvantage, which is a drastic detoriation in mechanical properties. Their average tensile and flexural strengths are 65.5 and 102.8 MPa, which is approximately 10.7 and 24.8 MPa less than in the case of off-stoichiometric formulations containing 1,6-bis­(2,3-epoxypropoxy)­hexane.^40^ This is due to the increased brittleness of the highly cross-linked structure, which does not provide suitable conditions for close packing,^74^ making the material less resistant to applied stress.^73^ In the end, it is worth noting that replacing the diluent does not ensure a clear separation of the two curing stages, which again leads to quasi-sequential curing. If a clearer separation is necessary, a different combination of anhydride and imidazole should be searched and investigated.

## Supplementary Material



## Notes

Curing conditions of final off-stoichiometric materials (Table S1); DSC data of DG80DD20-ANHYz reactive (Table S2), intermediate (Table S3) and final materials (Table S4); changes in viscosity during the activation of the second curing stage for chosen off-stoichiometric samples (Figure S1); DMA data of DG80DD20-ANHYz final materials (Table S5) and of DG80DD20-ANHY100 final materials cured under various curing conditions (Table S6); TGA data of DG80DD20-ANHYz final materials (Table S7), FTIR spectra of reactive (a), precured (b), further cured at 100 °C (c) and final (d) DG80D20-ANHYx samples (combined epoxy-anhydride band) (Figure S2), (anhydride and ester bands (Figure S3) and (hydroxyl band) (Figure S4) (PDF)
