# Supplementary material for: Sequential Diluted Dual-Curable Epoxy-Anhydride Thermosets: Effect of Epoxy-Based Reactive Diluent on Curing Behavior, Thermal and Mechanical Properties
Source: ACS Omega. 2026 Jul 22;11(30):45450–64. doi: 10.1021/acsomega.6c04947 (PMC13448948; doi:10.1021/acsomega.6c04947)
Supplement: Supplementary file 1 [file ao6c04947_si_001.pdf]

Supporting Information:

Sequential Diluted Dual-Curable Epoxy-Anhydride Thermosets: Effect of Epoxy-Based Reactive Diluent on Curing Behavior, Thermal and Mechanical Properties

Anna M. Dlugaj[1]**, David May*[1,2]

AUTHOR ADDRESS: [1]Leibniz-Institut für Verbundwerkstoffe GmbH, Erwin-Schröndiger 58, 67663 Kaiserslautern, Germany; [2]Faserinstitut Bremen e.V., University of Bremen, 28359 Bremen, Germany

*Email: Anna.Dlugaj@ivw.uni-kl.de

SAMPLE PREPARATION

The curing conditions of final off-stoichiometric DG80DD20-ANHYz materials are shown in Table S1.

Table S1. Curing conditions of final off-stoichiometric materials.

Formulation	Pre-curing time at 45°C (h)	Curing time at 100°C (h)	Post-curing time at 150°C (h)
DG80DD20-ANHY40	10	1	7
DG80DD20-ANHY42.5	10	1	7
DG80DD20-ANHY45	12	1	7

CURING BEHAVIOR

The DSC data of reactive, intermediate and final DG80DD20-ANHYz materials are shown in Table S2, Table S3 and Table S4, respectively.

Table S2: DSC data of DG80DD20-ANHYz reactive materials.

Formulation	T_{g0}^{DSC} (°C)	$T_{p1,r}^{DSC}$ (°C)	$T_{p2,r}^{DSC}$ (°C)	ΔH_{1r}^{DSC} (J g^{-1})	ΔH_{2r}^{DSC} (J g^{-1})	ΔH_{3r}^{DSC} (J g^{-1})	T_{g1}^{DSC} (°C)
DG80DD20-ANHY40	-44.5	126.2	139.2	174.9	173.6	2.3	100.3
DG80DD20-ANHY42.5	-44.8	127.5	140.1	180.7	162.6	2.5	100.6
DG80DD20-ANHY45	-45.0	128.9	141.9	190.2	145.3	2.6	99.8
DG80DD20-ANHY100	-50.1	143.8	173.5	369.9	30.3	-	113.8

Table S3: DSC data of DG80DD20-ANHYz intermediate materials.

Formulation	T_{gi}^{DSC} (°C)	T_{gi}^{DSC} (°C)	T_{gi}^{DSC} (°C)	T_{gi}^{DSC} (°C)	T_{gi}^{DSC} (°C)	T_{gi}^{DSC} (°C)
Pre-curing time	9 h	10 h	11 h	12 h	13 h	14 h
DG80DD20-ANHY40	6.9	9.2	9.7	10.2	-	-
DG80DD20-ANHY42.5	5.3	8.3	11.9	14.3	-	-
DG80DD20-ANHY45	-	-	15.3	17.3	19.1	19.4
Formulation	$T_{p,i}^{DSC}$ (°C)	$T_{p,i}^{DSC}$ (°C)	$T_{p,i}^{DSC}$ (°C)	$T_{p,i}^{DSC}$ (°C)	$T_{p,i}^{DSC}$ (°C)	$T_{p,i}^{DSC}$ (°C)
Pre-curing time	9 h	10 h	11 h	12 h	13 h	14 h
DG80DD20-ANHY40	137.2	129.6	129.6	132.9	-	-
DG80DD20-ANHY42.5	137.0	132.6	132.6	134.6	-	-
DG80DD20-ANHY45	-	-	130.3	130.9	131.2	139.2
Formulation	ΔH_{i}^{DSC} (J g^{-1})	ΔH_{i}^{DSC} (J g^{-1})	ΔH_{i}^{DSC} (J g^{-1})	ΔH_{i}^{DSC} (J g^{-1})	ΔH_{i}^{DSC} (J g^{-1})	ΔH_{i}^{DSC} (J g^{-1})
Pre-curing time	9 h	10 h	11 h	12 h	13 h	14 h
DG80DD20-ANHY40	156.1	153.4	165.5	153.2	-	-
DG80DD20-ANHY42.5	187.1	161.2	147.1	144.9	-	-
DG80DD20-ANHY45	-	-	141.8	147.8	119.9	147.5

Table S4: DSC data of DG80DD20-ANHYz final materials.

Formulation	T_{gf}^{DSC} (°C)	T_{gf1}^{DSC} (°C)	T_{gf2}^{DSC} (°C)
Curing conditions	1 h 100 °C	1 h 100 °C & 7 h 150°C	1 h 100 °C & 7 h 150°C
DG80DD20-ANHY40	105.1	134.1	131.0
DG80DD20-ANHY42.5	107.9	135.2	131.9
DG80DD20-ANHY45	110.1	136.0	132.4

The changes in viscosity during the activation of the second curing stage for samples that showed a slight (left column) and no shoulder (right column) in the DSC curves of intermediate materials are shown in Figure S1.

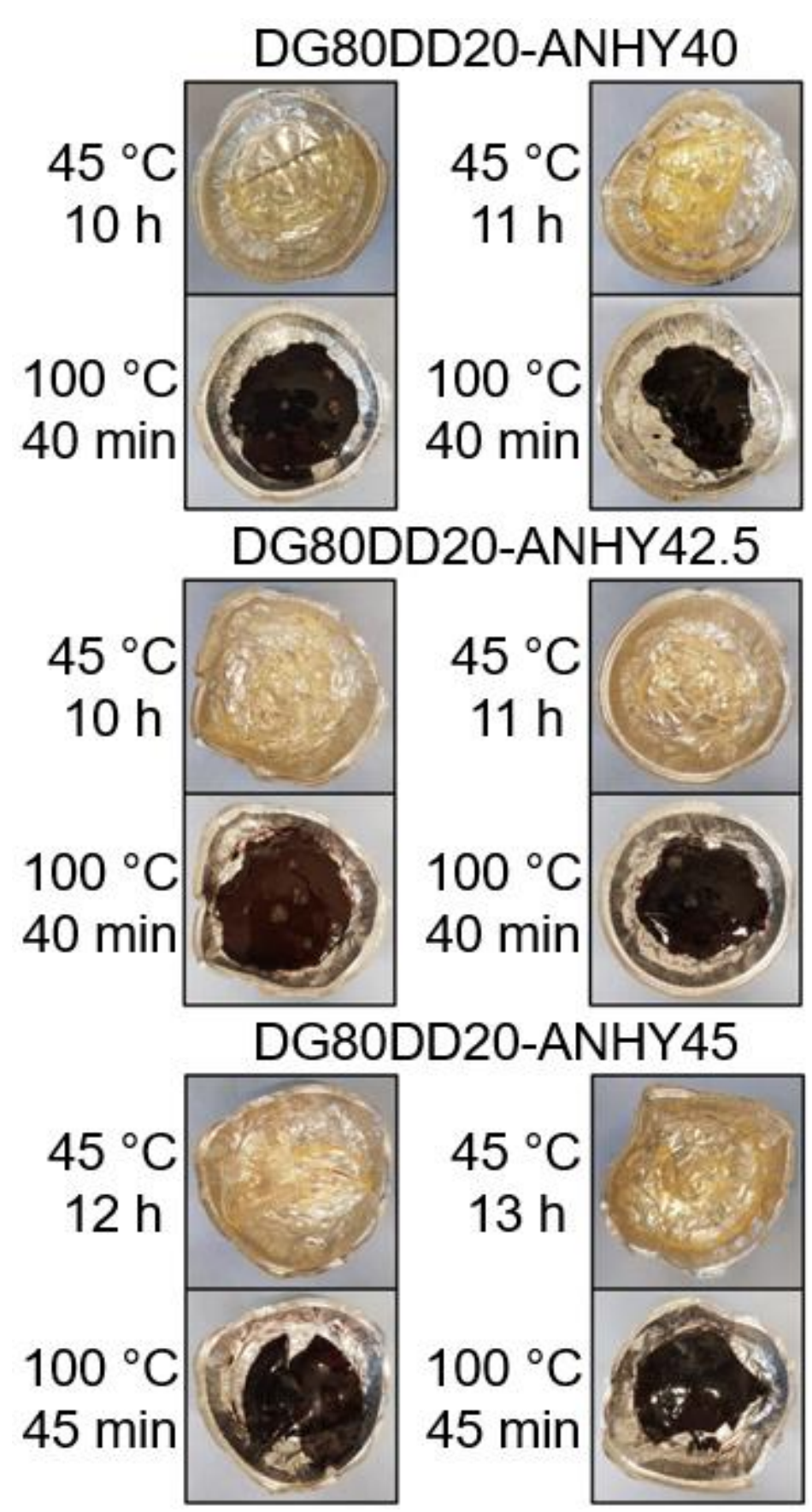

Figure S1: Changes in viscosity during the activation of the second curing stage for chosen off-stoichiometric samples.

THERMOMECHANICAL PROPERTIES

The DMA data of DG80DD20-ANHYz final materials are shown in Table S5, and of DG80DD20-ANHY100 final materials cured under different curing conditions in Table S6, respectively.

Table S5: DMA data of DG80DD20-ANHYz final materials.

Formulation	T_{gf1}^{DMA} (°C)	$v_{e,f1}^{DMA}$ (mol cm^{-1})	$\Delta(E', E'')_1$ (MPa)	$FWHM_1$ (°C)	M_{c1}^{DMA} (g mol^{-1})	$E'(0°)_1$ (GPa)
DG80DD20-ANHY40	137.6	0.00448	52.6	16.0	259.6	2.4
DG80DD20-ANHY42.5	137.2	0.00423	49.7	15.1	275.2	2.5
DG80DD20-ANHY45	136.6	0.00409	47.8	15.5	284.7	2.4
DG80DD20-ANHY100	122.9	0.00182	21.5	15.4	645.9	2.8
Formulation	T_{gf2}^{DMA} (°C)	$v_{e,f2}^{DMA}$ (mol cm^{-1})	$\Delta(E', E'')_2$ (MPa)	$FWHM_2$ (°C)	M_{c2}^{DMA} (g mol^{-1})	$E'(0°)_2$ (GPa)
DG80DD20-ANHY40	137.3	0.00432	50.8	15.9	269.3	2.6
DG80DD20-ANHY42.5	136.1	0.00405	47.5	15.4	287.4	2.7
DG80DD20-ANHY45	135.7	0.00393	46.0	15.6	296.3	2.6
DG80DD20-ANHY100	125.6	0.00166	19.8	14.0	708.2	3.0

Table S6: DMA data of DG80DD20-ANHY100 final materials cured under various curing conditions.

DG80DD20-ANHY100 sample after different curing conditions	T_{gf1}^{DMA} (°C)	$v_{e,f1}^{DMA}$ (mol cm^{-1})	$\Delta(E',E'')_1$ (MPa)	$FWHM_1$ (°C)	M_{c1}^{DMA} (g mol^{-1})	$E'(0°)_1$ (GPa)
4 h 80°C & 2 h 130°C	122.9	0.00182	21.5	15.4	645.9	2.8
4 h 80°C & 7 h 150°C	125.5	0.00183	19.6	15.3	642.4	2.6
4 h 80°C & 2 h 130°C & 7 h 150°C	125.5	0.00176	18.8	14.5	667.9	2.6
24 h 45°C & 1 h 100°C & 7 h 150°C	126.7	0.00195	20.9	14.3	602.9	2.7
Formulation	T_{gf2}^{DMA} (°C)	$v_{e,f2}^{DMA}$ (mol cm^{-1})	$\Delta(E',E'')_2$ (MPa)	$FWHM_2$ (°C)	M_{c2}^{DMA} (g mol^{-1})	$E'(0°)_2$ (GPa)
4 h 80°C & 2 h 130°C	125.6	0.00166	19.8	15.2	708.2	3.0
4 h 80°C & 7 h 150°C	126.3	0.00174	18.5	14.5	675.6	2.8
4 h 80°C & 2 h 130°C & 7 h 150°C	125.9	0.00162	17.2	12.6	725.7	2.8
24 h 45°C & 1 h 100°C & 7 h 150°C	126.9	0.00178	19.0	14.0	660.4	2.9

THERMAL STABILITY

The TGA data of DG80DD20-ANHYz final materials are shown in Table S7.

Table S7: TGA data of DG80DD20-ANHYz final materials.

Formulation	$T_{-5\,wt.-\%}$ (°C)	$T_{-50\,wt.-\%}$ (°C)	T_{max} (°C)
DG80DD20-ANHY40	366.4	430.1	426.5
DG80DD20-ANHY42.5	366.4	425.3	421.3
DG80DD20-ANHY45	364.1	424.2	419.2
DG80DD20-ANHY100	351.2	413.9	417.9

CURING MONITORING

The FTIR spectra of DG80DD20-ANHYz samples are shown in Figure S2, Figure S3 and Figure S4.

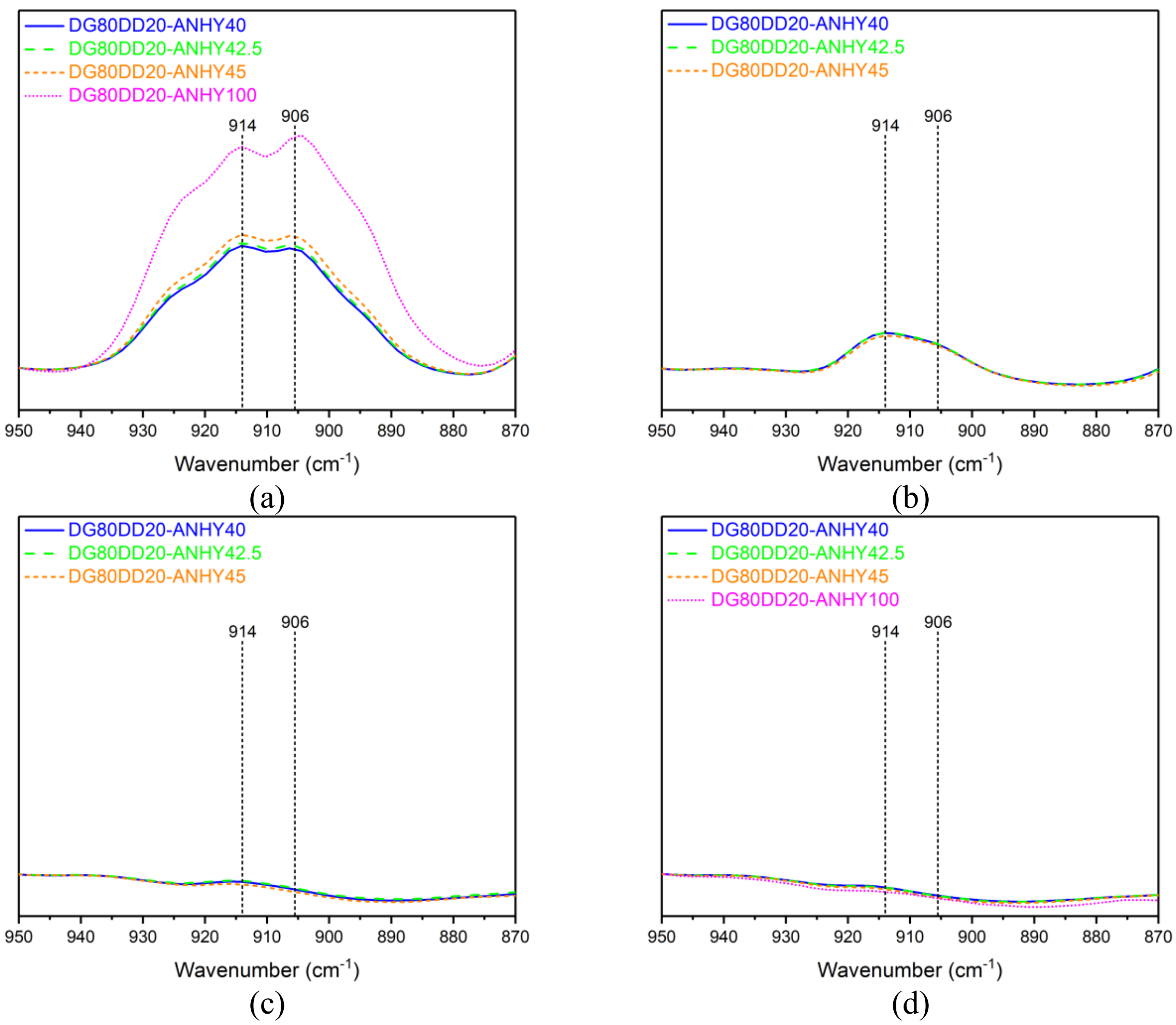

Figure S2: FTIR speactra of reactive (a), pre-cured (b), further cured at 100°C (c) and final (d) DG80DD20-ANHYx samples (combined epoxy-anhydride band).

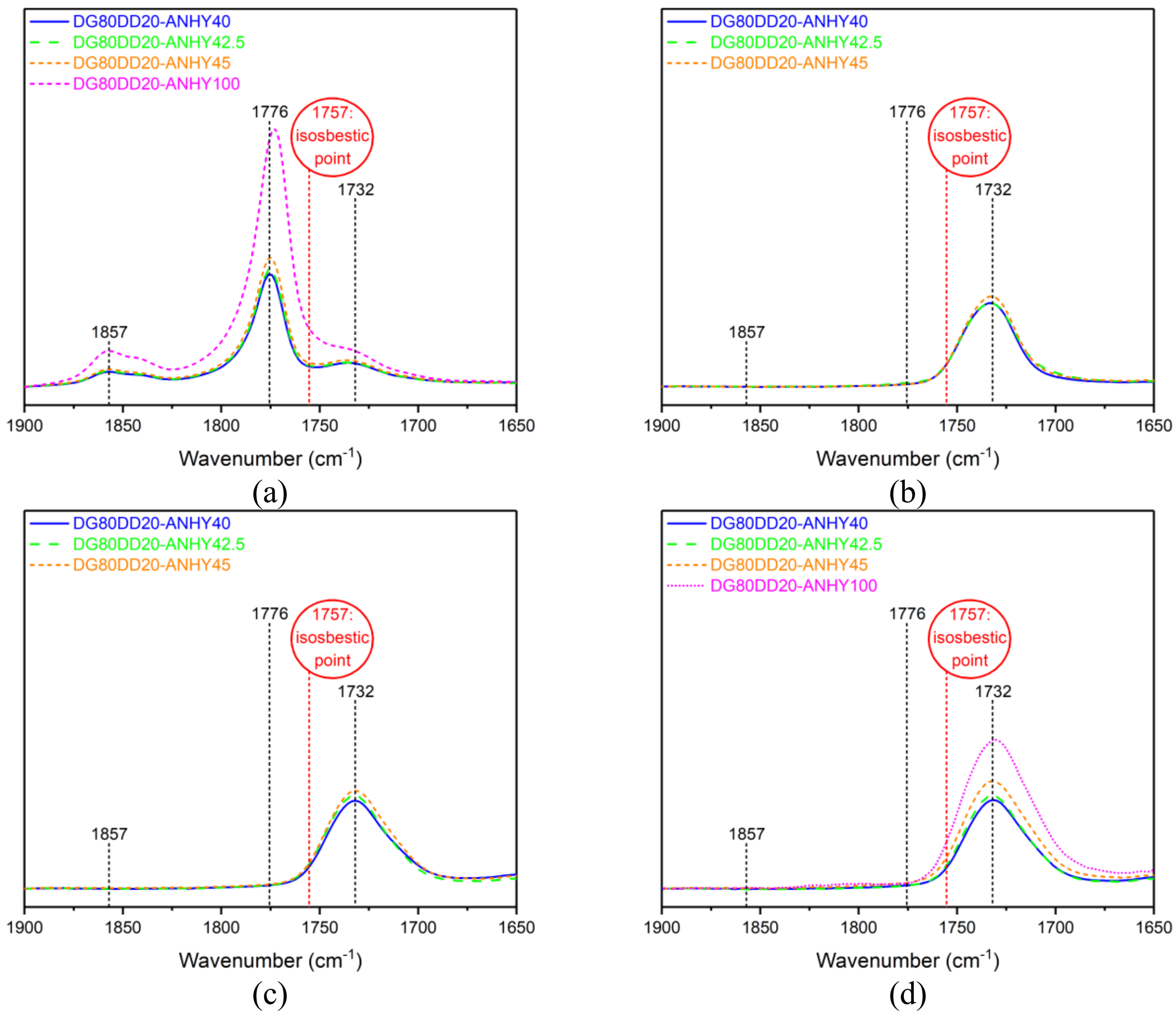

Figure S3: FTIR speactra of reactive (a), pre-cured (b), further cured at 100°C (c) and final (d) DG80DD20-ANHYx samples (anhydride and ester bands).

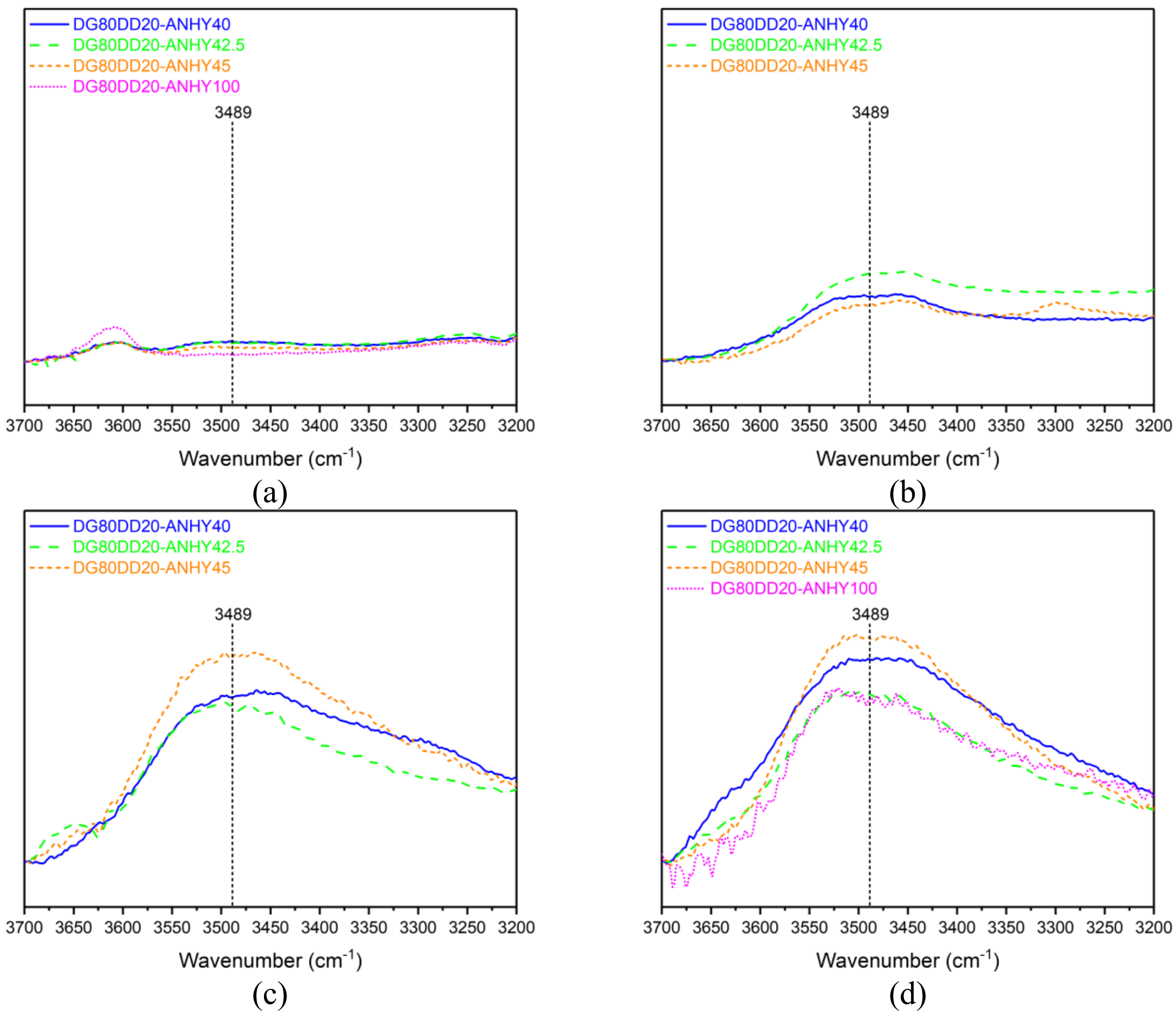

Figure S4: FTIR speactra of reactive (a), pre-cured (b), further cured at 100°C (c) and final (d) DG80DD20-ANHYx samples (hydroxyl band).
